# A pilot investigation of bovine schistosomiasis on Unguja Island, Zanzibar, raises a new concern for elimination of urogenital schistosomiasis

**DOI:** 10.1186/s13071-025-06698-y

**Published:** 2025-03-05

**Authors:** Shaali Ame, Othman Juma, Alexandra Juhász, Mtumweni Ali, Talib S. Suleiman, Geoffrey N. Gobert, Lucas J. Cunningham, Abigail Cawley, Lilly Atkins, Sam Jones, E. James LaCourse, Fatma Kabole, J. Russell Stothard

**Affiliations:** 1 Neglected Tropical Diseases Programme, Zanzibar Ministry of Health, Zanzibar, United Republic of Tanzania; 2 Zanzibar Livestock Research Institute, Ministry of Agriculture, Irrigation, Natural Resources and Livestock, Zanzibar, United Republic of Tanzania; 3 Department of Tropical Disease Biology, Liverpool School of Tropical Medicine, Liverpool, L3 5QA UK; 4 Institute of Medical Microbiology, Semmelweis University, Budapest, 1089 Hungary; 5 School of Biological Sciences, Queen’s University Belfast, Belfast, BT9 5DL UK

**Keywords:** One Health, *Schistosoma bovis*, *Schistosoma mattheei*, Urogenital schistosomiasis, Hybrids, Molecular xenomonitoring, *Bulinus forskalii* sp.

## Abstract

Our pilot parasitological investigation of cattle, supplemented with molecular DNA characterisation of encountered schistosomes, sheds first light upon bovine schistosomiasis on Unguja Island, Zanzibar. During February 2024, a total of 99 cattle were examined. Of these, 47 were exported animals from the Tanzanian mainland, designated for slaughter at two governmental abattoirs (Kisakasaka and Muwanda), and 52 were free-grazing animals sampled from four grazing locations within the island’s North and West-B regions. Upon visual inspection of 31 cattle carcasses at Kisakasaka for adult worms, the prevalence of bovine schistosomiasis was 51.6%; however, upon faecal miracidia hatching test (MHT) it was 80.6%. At Muwanda, only faecal MHT was used, finding a much lower prevalence of 12.5%. In free-grazing animals, the prevalence of bovine schistosomiasis by MHT was 0.0%. At Muwanda, the animal quarantine paddock was in disrepair, inclusive of a large pond now acting as a watering point. Here, numerous *Bulinus forskalii* sp. were found. Whilst no snails were observed to shed schistosome cercariae, molecular xenomonitoring did detect a pre-patent infection prevalence of 10.8%, with *Schistosoma bovis* firmly incriminated. Molecular DNA characterisation of adult schistosomes (*n* = 19) by real-time polymerase chain reaction (PCR) and high-resolution melt profiling, alongside DNA sequencing, also identified *S. bovis*, although two worms were putative *S. bovis-S. mattheei* hybrids. Atypical intrauterine eggs of *S. bovis* were noted upon microscopy of a worm pair. A broader screen of 92 miracidia confirmed *S. bovis* and three miracidia as *S. bovis-S. mattheei* hybrids. Contrasting with Pemba Island, Zanzibar, where autochthonous transmission of *S. bovis* can occur, bovine schistosomiasis on Unguja Island currently appears restricted to imported animals alone. However, the seminal detection of putative *S. bovis-mattheei* hybrids, alongside the current inadequate quarantine facilities at Muwanda, raises a new concern that such hybrid schistosomes may escape and enter the island’s hinterland. Should this happen, surveillance and control of urogenital schistosomiasis on Unguja would be compromised and further complicated. We therefore strongly recommend immediate repair and improved maintenance of governmental animal quarantine facilities. Future epidemiological surveys of imported cattle are now well justified, not only to better understand the full repertoire of hybrid schistosomes present but also to develop appropriate mitigating interventions.

**Graphical Abstract:**

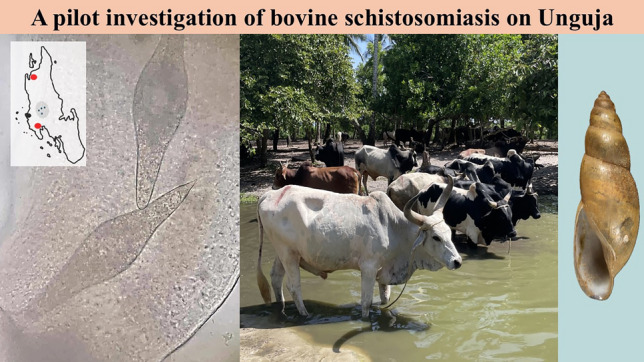

**Supplementary Information:**

The online version contains supplementary material available at 10.1186/s13071-025-06698-y.

Urogenital schistosomiasis (UGS) is a neglected tropical disease (NTD), endemic across much of continental Africa, and is mainly caused by the trematode blood fluke *Schistosoma haematobium* [1]. Autochthonous transmission of UGS occurs on both islands of Zanzibar, Unguja and Pemba, where long-standing efforts in disease control have forged real prospects in local elimination as a public health problem [2–4]. A contributing favourable factor has been the notable absence of hybrid schistosomes, particularly those between *S. haematobium* and *Schistosoma bovis*, as a recent genomic investigation of *S. haematobium* isolates from Zanzibar confirms they are amongst the purest form of this species [5], though mitochondrial DNA barcoding and DNA microsatellite analyses have previously revealed surprising populational diversity [6, 7]. *Schistosoma bovis* is a common parasite of livestock, cattle in particular, and is broadly found within the northern tropical belt of Africa [8]. Of growing public health concern are *S. haematobium*-*bovis* hybrids that can be found outside this region [9], for example, northwards on the Mediterranean island of Corsica and southwards in Malawi [10, 11]. Owing to their increased zoonotic potential, molecular surveillance of hybrid schistosomes within livestock is being advised, adopting a more One Health approach where appropriate, to help safeguard current gains in UGS control [1, 12, 13].

Across Zanzibar, surveillance of bovine schistosomiasis is still nascent [2]. In 2016 *S. bovis* was first noted, using molecular methods of identification of emergent cercariae from an intermediate host snail *Bulinus globosus*, at Kinyasini, Pemba [14, 15]. This species, together with *Bulinus nasutus*, is placed within the *Bulinus africanus* group. These two species have an allopatric distribution, save for one location on Pemba [15], with *B. globosus* being the major intermediate host for *S. haematobium* [2]. Then, in 2019, several cattle grazing near Kinyasini, Pemba, were confirmed to be infected using egg-detection coproscopy [16]; a later DNA characterisation of collected cercariae and miracidia with the mitochondrial cytochrome oxidase subunit I (*cox*1) gene, upon phylogeographical analysis, pointed towards an off-island origin [15, 17]. Unlike *S. haematobium*, it was inferred that *S. bovis* was introduced to Pemba, most likely from imported mainland cattle [2]. Importation of cattle, which follows the guidelines of the United Republic of Tanzania's Animal Resources Management Act of 1999 (https://www.fao.org/faolex/results/details/en/c/LEX-FAOC172321/), is increasing in response to a growing demand for fresh meat from an expanding local tourism industry. The latter is the island’s main source of economic revenue today (https://www.ocgs.go.tz/tourism). Further analysis of snail collections for pre-patent infections with a molecular xenomonitoring assay indicated a wider distribution of *S. bovis* on Pemba [17]. Despite a concurrent increase in imported cattle onto Unguja, primarily for human consumption, *S. bovis* has yet to be detected [2]. This could be due, in part, to an absence of formal surveillance for bovine schistosomiasis in abattoirs on Unguja.

To make an initial assessment on the putative occurrence of bovine schistosomiasis on Unguja, a pilot parasitological investigation of imported and local cattle was conducted in February 2024. This investigation was augmented with molecular identification of retrieved schistosome life cycle stages, inclusive of collected adult worms (from mesenteric veins and sliced livers from carcasses), hatched miracidia from faeces and genotyping pre-patent infections in field-caught snails by molecular xenomonitoring [18, 19]. A selection of cattle designated for slaughter (*n* = 47), and ascertained as most likely to have an off-island origin upon discussions with abattoir staff, was inspected at two governmentally administered island abattoirs, West-B Region–Kisakasaka (*n* = 31) at [−5.910706°, 39.225595°] and North Region–Muwanda (*n* = 16) at [−6.257599°, 39.279135°]; see Fig. 1. A comparable number of free-grazing animals (*n* = 52) in areas within North and Central Unguja, where *B. globosus* is common, was inspected by coprological methods alone. Here, fresh faecal samples were obtained from cattle at Dole-Kianga (*n* = 12) at [−6.115393°, 39.258432°], Mgeni Haji/Kwamba (*n* = 14) at [−6.093509°, 39.315278°], Kidimni (*n* = 11) at [−6.130715°, 39.297797°], and Koani (*n* = 15) at [−6.132732°, 39.293299°] (Fig. 1). All faecal materials, approximately 15 g per animal, were processed by miracidia hatching test (MHT) to ascertain the animal’s infection status [10].

**Fig. 1:**
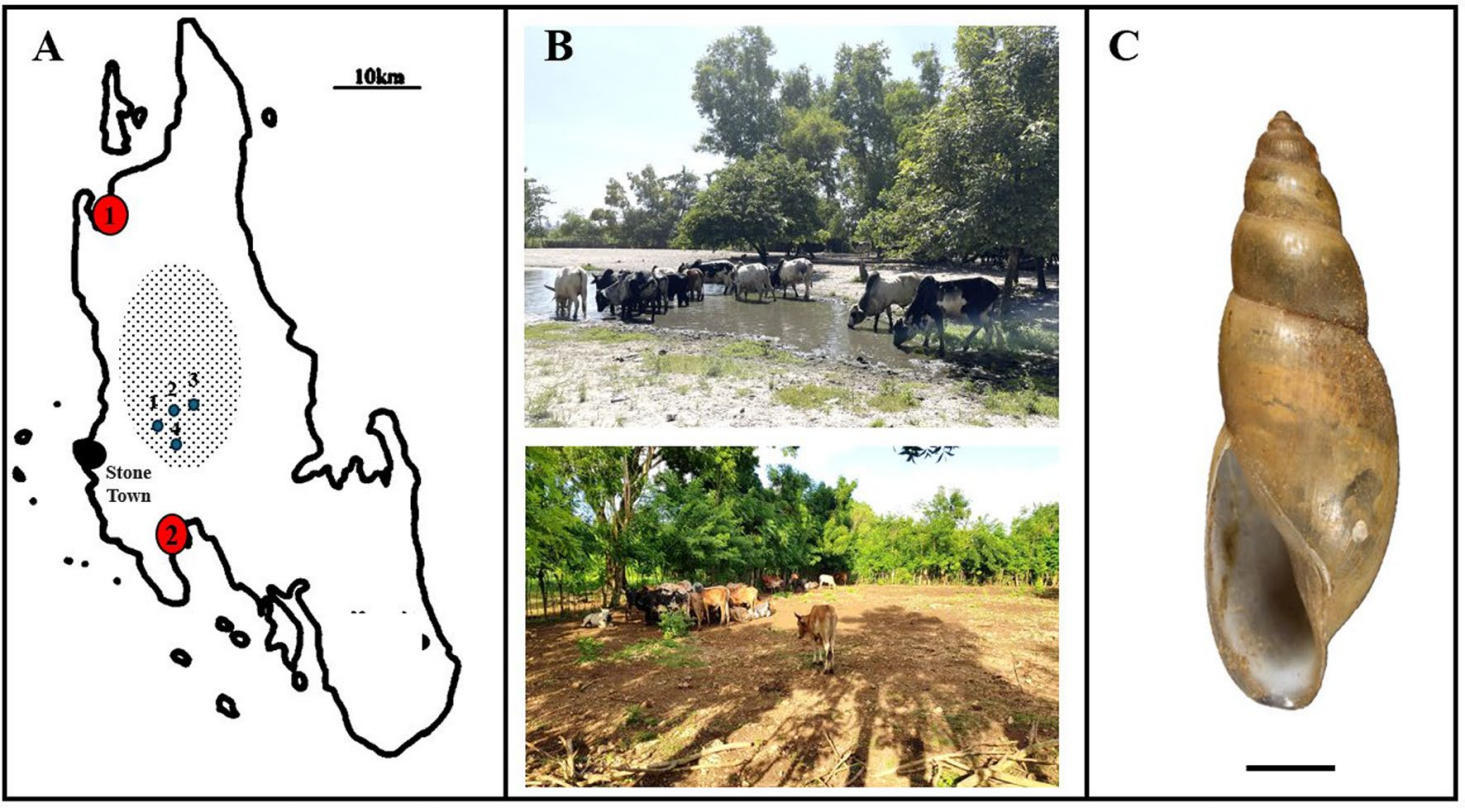
The outline area where this pilot investigation took place, with pertinent photographs of the quarantine areas of each abattoir and a typical *Bulinus forskalii* sp. snail encountered at Muwanda abattoir quarantine holding area. **A** A sketch map of the sampling locations: red circle 1—Muwanda abattoir, red circle 2—Kisakasaka abattoir, and the four blue dots, 1–4, where field grazing cattle faecal sampling took place, 1—Dole-Kianga, 2—Mgeni Haji/Kwamba, 3—Kidimni, and 4—Koani within the central region (shaded area) where *Bulinus globosus* can be found; **B** On-site photos of quarantine areas where cattle were held in each abattoir for 2–3 days before slaughter: Muwanda (*top*) and Kisakasaka (*bottom*). **C** A typical shell of *B. forskalii* sp. snail (shell scale bar: 2 mm) found in large numbers around the drinking pond within Muwanda quarantine paddock

It is worth mentioning that at each abattoir, cattle were off-loaded from locally beached dhows, then walked under supervision, typically defecating along the way, the short distance (~ 1 km) to a designated quarantine paddock, with secure metal fencing and a water trough. These paddocks act as short-term (~2–3 days) holding areas before animals are again walked on for *dhabah*, i.e. halal slaughter, which takes place late in the evening, with meats collected by several vendors early in the morning. Both abattoir facilities became fully operational in 2017, and today each processes some 7500 animals annually. From specific observation of poor water, sanitation and hygiene (WASH) conditions within animal holding facilities in Muwanda, where cattle were seen watering within their quarantine paddock from a large pond as the metal drinking trough was dysfunctional, and upon a fingertip search of the pond’s edge, numerous *B. forskalii* group species were found (Fig. 1). All collected snails (*n* = 75) were later screened, upon exposure to artificial light, for shedding schistosome cercariae under view of a dissecting microscope. No schistosome cercaria was seen, but cercariae of rumen fluke were noted originating from two snails. A selection of these snails (*n* = 46), as placed in absolute ethanol, was later examined by molecular xenomonitoring using a genus-specific TaqMan probe [20], to reveal five snails (10.8%) with strong DNA evidence of *Schistosoma* spp. infection. Following Cunningham et al. [18], upon real-time polymerase chain reaction (rtPCR) high-resolution melt (HRM) typing analyses [18], four snails harboured *S. bovis*, while the single remainder likely harboured *S. haematobium*. These snails were also typed with molecular *cox*1 DNA barcoding to elucidate their precise species identity following generic molecular taxonomic methods for *Bulinus* [20]. Upon National Center for Biotechnology Information (NCBI) Basic Local Alignment Search Tool (BLAST) search, the obtained *cox*1 matched with 100% query cover and percentage identity of ~99.4% *B. forskalii* sp. from Pemba (Accession no. AM921832.1). The snails within Muwanda paddock were not *B. forskalii* but rather its morphologically cryptic sister species currently recognised as *B. forskalii* sp. and presented a new additional location of its presence on Unguja [2]. The compatibility of this taxon with schistosomes is presently not known and awaits experimental challenge.

Across two working evenings at the Kisakasaka abattoir, 35 cattle carcasses were examined immediately after slaughter for schistosomes by visual inspection of mesenteric veins and sliced livers. Adult worms were retrieved either by hand using fine forceps (from mesenteric veins) or by filtration across a metal 212 μm mesh sieve (from sliced livers), using previously described methods [10]. Worms were then placed into normal saline for later viewing under a dissecting microscope (×10–×40), before female worms were more closely inspected under a compound microscope (×400) to view intrauterine egg morphology, followed by separation from their male partners. Each worm was then stored individually in absolute ethanol for subsequent identification with molecular DNA analyses. The prevalence of bovine schistosomiasis by carcass inspection was 51.6%, with an average number of 12 worms per animal and a maximum of 32 observed (Table 1). With one exception, all female worms inspected contained eggs typical of *S. bovis* morphology, displaying prominent polar spindles with an obvious equatorial bulge. One adult female was noted to contain atypical *S. bovis*-shaped eggs, appearing without an equatorial bulge, being more rhomboid/diamond-shaped and slightly shorter in total length, at ~180 µm; see Fig. 2. Whilst in the abattoir, a cursory inspection of several cattle rumens noted the presence of numerous rumen flukes (Supplemental Fig. 1).

**Table 1:** Prevalence of bovine schistosomiasis by detection of adult worms by carcass inspection

Abattoir	Cattle sampled		Schistosome worms observed	
	*N*	No. positive	Mean worm burden (min–max)	Prevalence (%) (95% CI)
Kisakasaka	31	16	12 (1–32)	51.6 (33.1–69.8)

**Fig. 2:**
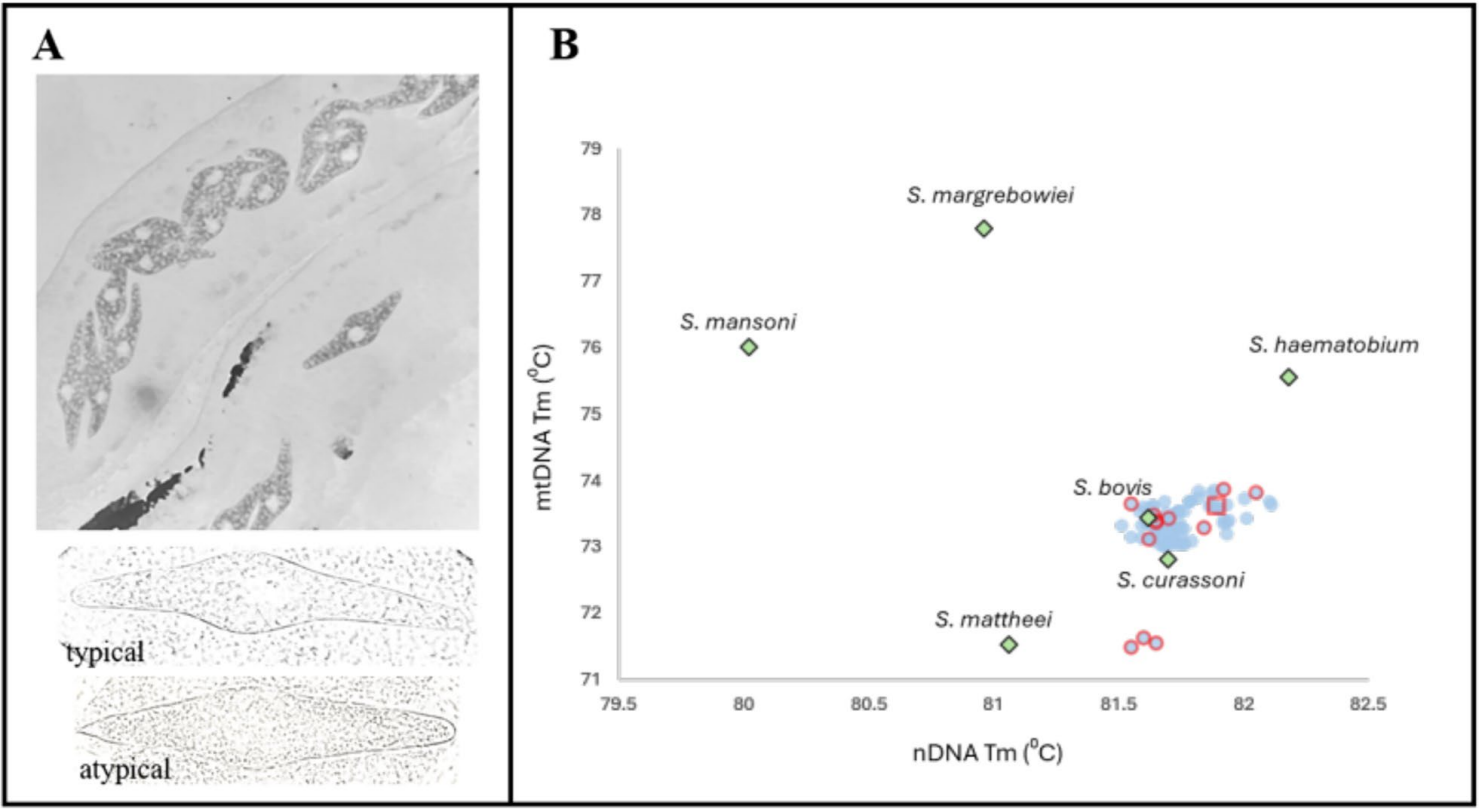
Characterisation of schistosomes by egg morphology and identification by DNA HRM typing. **A** Representative view (×100) of numerous intrauterine eggs within the worm’s oviduct (top image), with an example of the two distinctive morphotypes of intrauterine eggs—typical egg with prominent equatorial bulge and an atypical egg without equatorial bulge, more rhomboid in shape and slightly shorter in length, seen at ×400. From later DNA profiling, the atypical egg was likely a *S. bovis-mattheei* hybrid. **B** Scatter plot of mitochondrial DNA (mtDNA) and nuclear DNA (nDNA) HRM temperatures with expected species plot locations indicated with the green diamonds (and species names) and adult worm and miracidial blue circles. Red circles indicate samples that were selected for sequencing, red squares indicate samples that produced a usable sequence. The isolated red squares marked i and ii are adult worm profiles sent for DNA sequencing

For molecular identification purposes, 19 adult worms underwent DNA extraction (11 males and eight females) and rtPCR HRM analysis following Cunningham et al. [18]. Plotting the melting temperature (Tm) of the obtained nuclear and mitochondrial amplicons identified 17/19 as *S. bovis*, although two samples (i and ii) were placed outside this grouping; see Fig. 2B. Following the rtPCR HRM, DNA sequences from 10 representative worms for the *cox*1 and ribosomal internal transcribed spacer (ITS) gene were obtained. Upon NCBI BLAST search for *cox*1, nine matched 99.0% query cover and 99.6–99.8% identity with *S. bovis* from Pemba, Zanzibar (Accession no. OK484569) and Kisumu, Kenya (Accession no. FJ897160), while the remainder contained multiple mixed single-nucleotide bases which matched closely with polymorphisms between *S. bovis* and *S. mattheei*. This strongly suggests that this individual worm was a hybrid or similar introgression between two schistosome species. Of the 10 ITS sequences, nine matched with 100% query cover and 100% identity with *S. bovis* (Accession no. OK447652). Unfortunately, the ITS sequence from the putative *cox*1 hybrid was not of sufficient quality for an unambiguous comparative analysis.

Supplemental to carcass inspection, faecal samples were collected from the intestine of slaughtered animals at Kisakasaka or immediately after defecation from living cattle, either in the quarantine paddock at Muwanda or free-grazing in the North and Central areas of the island, Table 2. Faecal samples were analysed using a modified MHT to estimate the prevalence and intensity of infection [10]; approximately 15 g of each faecal sample was processed. If miracidia were seen during a 4-min period of observation, the sample was deemed positive. Intensity of infection was estimated upon harvesting each observed miracidium by micropipette in 2.5 µl of water onto Whatman FTA^®^ cards (GE Healthcare Life Sciences, Amersham, UK), thereby keeping a tally of numbers. In total, 178 miracidia were obtained in this manner. The prevalence of bovine schistosomiasis by MHT was 80.6% at Kisakasaka, 12.5% at Muwanda and 0.0% within the North and Central regions. The mean prevalence of bovine schistosomiasis across the two abattoirs was 57.4%, with the intensity of infections at Kisakasaka some threefold higher than at Muwanda, Table 2. In contrast to the absence of infections within cattle in the North and Central regions, it is immediately apparent that bovine schistosomiasis within imported cattle is common.

**Table 2:** Prevalence and intensity of bovine schistosomiasis on Unguja

Sampling location	Cattle		Miracidia	Prevalence
	*N*_MHT_^a^	No. positive	Mean no.^b^	(%) (95% CI)
Kisakasaka	31	25	6.3	80.6 (62.5–92.5)
Muwanda	16	2	2.0	12.5 (1.6–38.4)
Total from abattoir	47	27	4.2	57.4 (42.1–71.7)
Central grazing area	52	0	na	0.0 (0.0–5.8)
Total cattle examined	99	27	na	27.3 (18.8–37.2)

na = not applicable

^a^ MHT—miracidia hatching test

^b^ The count of observed miracidia over a 4-min period, with miracidia removed by pipette upon first observance

To gain additional insight into the schistosome species identity, rtPCR HRM assays were attempted on all miracidia stored on FTA cards after alkaline DNA extraction. This resulted in 92 miracidia that could be typed using both nuclear and mitochondrial loci (Fig. 2B). The Tm profiles of 89/92 samples clustered with *S. bovis*, while three samples produced melt profiles that clustered with *S. bovis* nuclear DNA and *S. mattheei* mitochondrial DNA melt signatures, indicating a putative *S. bovis*-*mattheei* hybrid. It should be noted that these three putative hybrids came from the same animal. To confirm HRM findings, amplification of *cox*1 and ITS from the three putative hybrid samples plus an additional 10 putative *S. bovis* samples was attempted. However, only four samples produced PCR products sufficient for DNA sequencing, and the quality was poor such that only a partial ITS was obtained, but this matched, with 100.0% query cover and 99.1%, *S. bovis* (Accession no. OK447652).

Collectively, these pilot findings shed first light on bovine schistosomiasis on Unguja Island and raise a new public health concern that may complicate and even confound future efforts for the elimination of UGS from Zanzibar. The most significant—and totally unexpected—finding was the detection of a *S. bovis-mattheei* hybrid. This brings together observations on intrauterine egg morphology, alongside those on genotypes of both adult worms and miracidia (Fig. 2). Historically, although experimental crosses between *S. bovis* and *S. mattheei* can yield viable progeny, albeit to F3 only [21], this is the first time, to our knowledge, that *S. bovis-mattheei* has been encountered in nature. A wider concern is that it is currently unknown what the definitive or intermediate host range of this hybrid variant may be and, if given an opportunity, how these worms may or may not interact with *S. haematobium* [22]. It is certain, however, that this infection in cattle was acquired off-island, most likely at mixed transmission foci within the Tanzanian mainland. It is believed, for example, that the most northern range of *S. mattheei* and the most southern range of *S. bovis* overlap here in Tanzania [23], though without further contemporary mapping information on the mainland this remains conjecture. Furthermore, as veterinary surveillance for bovine schistosomiasis is also scant, with only sporadic recent reports in the literature [24], it is difficult to ascertain whether this hybrid is currently restricted to transmission within livestock alone or already has, or in the future will, establish human transmission cycles. Nevertheless, it was reassuring to note that the more worrisome zoonotic *S. haematobium-bovis* variant was absent within this recent snapshot of schistosome diversity uncovered.

By inference from our pilot findings, it is particularly clear that imported cattle will continue to pose a tangible risk for the introduction of foreign schistosomes into Zanzibar. Given the scale of importation of cattle, which on Unguja alone would be in excess of 15,000 animals annually, and not forgetting those animals that enter under clandestine conditions, certain traditional intervention strategies are rendered either unaffordable or ineffective. Whilst the mainstay of public health control of UGS in people is by large-scale administration of the anthelminthic praziquantel, such human drug stocks are donated gratis [1]. By contrast, no such stocks exist for treatment of bovine schistosomiasis; hence the obvious strategy of animal treatment at port of exit or port of entry is not possible. Furthermore, bovine schistosomiasis is not included within the disease repertoire of the United Republic of Tanzania’s Animal Resources Management Act of 1999. Amongst all available alternatives, the most feasible current intervention is strict adherence to on-island quarantine and isolation measures, with animals sent on to slaughter as quickly as possible, thereby minimising any environmental water contact. We have observed, however, that upkeep of these imported animals’ WASH provision is unsatisfactory within their short-term storage paddock at Muwanda.

The WASH situation at Muwanda is of foremost concern, as potential intermediate host snails were in very close proximity to imported cattle. From our molecular xenomonitoring analysis, there is ample evidence of miracidial exposure, such that it was simply a matter of good fortune that only *B. forskalii* sp. was present and not another intermediate snail species with greater schistosome/snail compatibility with miracidia shed from these animals. From previous laboratory infection challenge studies, *B. forskalii* is a competent intermediate host for *S. bovis* [25], and populations of this snail species are widespread across Zanzibar, sometimes sympatric with *B. africanus* group species [2]. It has been suggested that *Bulinus browni,* a *B. forskalii* group species more closely related to *B. forskalii* sp., may also be a competent natural intermediate host for *S. bovis* in Western Kenya [26]. All things considered, we recommend prompt repair of WASH facilities at Muwanda, with appropriate environmental modification of the paddock to render it free from all freshwater snails in the future. Prior to such environmental modification, perhaps upon improved sub-soil drainage, focal mollusciciding, as used elsewhere in Zanzibar [27], may be considered and would be suitable when cattle are temporarily vacant from this holding facility. Furthermore, all imported cattle should be strictly barred in their access to environmental watering sites that are shared with local animals, particularly when transferring from landing dhows to abattoir facilities.

As stated previously, autochthonous transmission of *S. bovis* takes place on Pemba [15], with *B. globosus* incriminated. Therefore, it would be reasonable to assume that future autochthonous transmission on Unguja is a strong possibility. In the past, a particularly advantageous feature in the control of UGS on Unguja has been the sole presence of *S. haematobium* but for how long will this continue if perennial transmission of *S. bovis* is ever established? The suspected changes in the future epidemiology of UGS now call for renewed effort and funding in environmental surveillance of both schistosomes and snails. Further targeted molecular epidemiological surveys of people and livestock would be an essential component. A firm conclusion from our pilot investigation, given the scale of cattle importation for the foreseeable future, is to first focus attention on surveillance of infections within imported cattle. This is to not only characterise the full repertoire of schistosomes present, hybrids in particular, but also gain a more comprehensive baseline One Health assessment that will underpin the development of appropriate future mitigating interventions. A key challenge will be developing suitable and affordable disease surveillance and control methods at scale.

## Supplementary Information

**Supplementary material 1.** Photograph of the internalsurfaces of a cattle rumen with numerous rumen flukes (indicated by white arrows).

## Data Availability

All Sanger sequence data generated were uploaded to the GenBank repository as described (*Schistosoma* *cox*1 sequences: GenBank accession numbers PQ878458–PQ878467; *Bulinus* *cox*1 sequences: GenBank accession numbers PQ878355–PQ878359.

## Abbreviations

BLAST: Basic Local Alignment Search Tool
*cox*1: Cytochrome oxidase subunit I
HRM: High-resolution melt
ITS: Internal transcribed spacer
NCBI: National Center for Biotechnology Information
NTD: Neglected tropical disease
PCR: Polymerase chain reaction
UGS: Urogenital schistosomiasis
WASH: Water, sanitation and hygiene
