# Supplementary figures and images for: A pilot investigation of bovine schistosomiasis on Unguja Island, Zanzibar, raises a new concern for elimination of urogenital schistosomiasis
Source: Parasit Vectors. 2025 Mar 5;18:89. doi: 10.1186/s13071-025-06698-y (PMC11883948; doi:10.1186/s13071-025-06698-y)

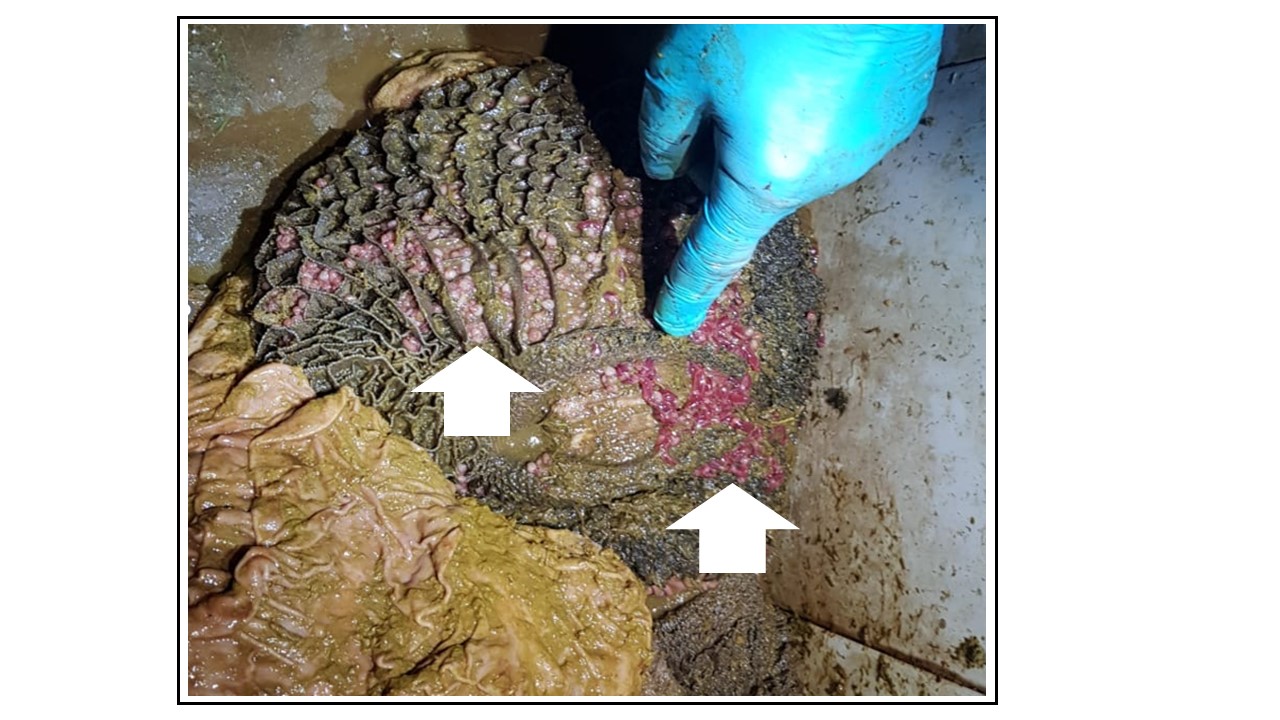

Supplement: Supplementary file 1 — Supplementary material 1. Photograph of the internalsurfaces of a cattle rumen with numerous rumen flukes (indicated by white arrows). [file 13071_2025_6698_MOESM1_ESM.jpg]
